# Association between age and surgical resection for patients with stage II or III colorectal cancer: national study

**DOI:** 10.1093/bjsopen/zrag071

**Published:** 2026-06-11

**Authors:** Adil Rashid, Helen Blake, Lu Han, Jan van der Meulen, Michael S Braun, Nicola S Fearnhead, Kate Walker

**Affiliations:** Department of Health Services Research and Policy, London School of Hygiene and Tropical Medicine, London, UK; Clinical Effectiveness Unit, Royal College of Surgeons of England, London, UK; Department of Applied Health Research, University College London, London, UK; Department of Health Services Research and Policy, London School of Hygiene and Tropical Medicine, London, UK; Clinical Effectiveness Unit, Royal College of Surgeons of England, London, UK; Department of Health Services Research and Policy, London School of Hygiene and Tropical Medicine, London, UK; Clinical Effectiveness Unit, Royal College of Surgeons of England, London, UK; Department of Oncology, The Christie NHS Foundation Trust, School of Medical Sciences, University of Manchester, Manchester, UK; Department of Colorectal Surgery, Cambridge University Hospital NHS Foundation Trust, Cambridge, UK; Department of Health Services Research and Policy, London School of Hygiene and Tropical Medicine, London, UK; Clinical Effectiveness Unit, Royal College of Surgeons of England, London, UK

**Keywords:** colorectal resection, frailty, performance status

## Abstract

**Introduction:**

Elderly patients with colorectal cancer (CRC) are less likely to undergo surgical resection. This study investigated the association between age and surgical resection, stratified by Eastern Cooperative Oncology Group performance status (PS), co-morbidities, tumour site, and mode of presentation.

**Methods:**

Using national data from 2017–2022, patients diagnosed with stage II–III CRC in the English National Health Service were identified. The primary outcome was receipt of surgical resection. Risk-adjusted rates of surgical resection by age were estimated using multilevel multivariable logistic regression, testing for interactions with PS, co-morbidities, tumour site, and mode of presentation. Between-hospital variation in rates of surgical resection was also examined.

**Results:**

Overall, 63 698 of 79 763 patients (79.9%) underwent surgical resection during the study period. PS substantially modified the relationship between age and surgery. After risk adjustment, approximately 90% of fit patients (PS 0 or 1) underwent surgery up to age 75 years, with a steep decline by age 90 years to 61.8% (95% confidence interval (c.i.) 58.3% to 65.3%) and 48.8% (95% c.i. 45.9% to 51.7%) for PS 0 and 1, respectively. For patients too unfit to work (PS ≥ 2), the adjusted rate of surgery was lower and declined earlier, from around 80% at age 60 years, and then more gradually to 25.7% (95% c.i. 23.5% to 27.8%) at age 90 years. After adjustment, between-hospital variation was larger in patients aged > 75 years than in younger patients (interquartile range 60.7–74.7% *versus* 84.9–90.7%, respectively).

**Conclusion:**

In patients fit enough to work, age is not associated with surgical decision-making until around age 75 years. In less fit patients, age is a key factor in decision-making across all ages. Substantial variation in rates of surgery between hospitals highlights the need for evidence-based guidelines to support equitable access to potentially curative treatment.

## Teaser

Among patients who are relatively fit (that is, those fully active or able to perform light work), surgery is performed in around 90% until the age of 75 years, with a steep drop above this age. In patients who are less fit (that is, would no longer be able to work), the rate of surgery declines from an earlier age, with around 80% undergoing surgery until age 60 years, followed by a more gradual decline in surgery rates. There is substantial between-hospital variation in the rate of major surgery after risk adjustment. This variation is more pronounced in patients older than 75 years.

## Introduction

Colorectal cancer (CRC) is one of the most common cancers in older adults^1^. In 2023, 35,243 patients were diagnosed with CRC in England, corresponding to an annual incidence of 62 per 100 000 people. Approximately one-quarter of these patients were aged ≥ 80 years^2^. In the National Health Service (NHS) in England, treatment decisions for patients with newly diagnosed CRC are coordinated by comprehensive multidisciplinary teams (MDTs)^3^. For stage I–III CRC, surgical resection is the primary curative treatment option. Local excision is an alternative strategy for select patients with stage I CRC^3,4^.

Balancing the risks of postoperative complications and mortality after surgical resection is complex in older CRC patients, with limited evidence from clinical trials, where older patients are under-represented^5^. Historically, older patients underwent surgical resection less often, with greater use of non-curative, less invasive treatment strategies^6–8^.

It has been suggested that less frequent use of surgical resection in older patients contributes to the poorer overall survival outcomes of CRC patients in England compared with other European nations^9^. Conversely, the lower use of surgical resection in older patients may reflect appropriate clinical decision-making given the higher prevalence of co-morbidities, poorer Eastern Cooperative Oncology Group (ECOG) performance status (PS), increased frailty, more advanced stage at diagnosis, and the higher rate of an emergency CRC diagnosis among older adults^8,10^.

Advances in perioperative care, minimally invasive surgical techniques, and preoperative optimization^11,12^ have led to substantial improvements in outcomes after CRC surgery in older adults^13,14^. As a result, it is increasingly recognized that decisions about surgical resection for newly diagnosed CRC patients should not be guided solely by age^3,15,16^. Clinical guidelines explicitly state that treatment recommendations for older adults should consider co-morbidities and measures of fitness, such as PS^3,17^.

This study aimed to use a unique, comprehensive national linked data resource to assess the association between age and surgical resection, stratified by PS, co-morbidities, tumour site, and mode of presentation, while also adjusting for other patient characteristics. In addition, the study aimed to investigate variation in surgical decision-making between hospitals. Understanding the interplay between age and measures of physical fitness in surgical decision-making, and the variation across hospitals, is an important step towards identifying targets to improve outcomes for older CRC patients.

## Methods

### Ethical considerations

This population-based cohort study was undertaken as part of the National Bowel Cancer Audit (NBOCA). The study was exempt from UK National Research Ethics Committee approval because it involved secondary analysis of an existing pseudonymized data set under Section 251 approval (CAG ECC 1-3(d)/2012).

#### Data sources

Individuals diagnosed with primary CRC in the English NHS during the 5-year study period (1 April 2017 to 31 March 2022) were identified in the NBOCA data set^2^. NBOCA holds a mandatory prospective database that includes patients diagnosed with primary CRC in the English NHS. These patients were linked to Hospital Episode Statistics (HES), an administrative data set of all hospital episodes in the English NHS^18^. The HES includes dates and types of admission, patient characteristics, discharge dates, Office of Population Censuses and Surveys Classification of Surgical Operations and Procedures, 4th revision (OPCS-4) procedure codes, and International Classification of Diseases, Tenth Revision (ICD-10) diagnosis codes.

#### Data variables

Data items in the NBOCA database include age at diagnosis, sex, date of diagnosis, diagnosing NHS hospital, date of surgical resection, tumour site (categorized as right-sided colon, left-sided colon, rectum), clinical and pathological staging, and ECOG PS (for the purpose of this study, PS was categorized as 0 (fully active), 1 (only able to carry out light or sedentary work), and ≥2 (no longer able to work or worse)). The clinical tumour, node and metastases (TNM) staging system (that is, staging based on information available at the time of diagnosis), was used to reflect tumour stage when treatment decisions were made (Union Internationale Contre le Cancer and the American Joint Committee on Cancer fifth edition^19,20^). For patients with missing clinical TNM data, pathological TNM stage (that is, staging also based on information available after surgical resection) was used when available. Cancer stage was grouped into four categories, with an additional category for missing stage^21^. Mode of presentation from NBOCA was categorized into elective (that is, referred by primary care physician or other healthcare professional/detected through screening) or emergency (diagnosed after an emergency admission). For patients with a missing mode of presentation in NBOCA, the mode of the first hospital admission in the HES with a CRC diagnosis was used instead^10^. ICD-10 codes in HES episodes were used to identify the number of co-morbidities recorded in the 2 years up to the date of the CRC diagnosis, according to the Royal College of Surgeons (RCS) Charlson co-morbidity score^22^ (with the number of co-morbidities categorized as 0, 1, and ≥ 2), as well as to determine frailty according to the Secondary Care Administrative Records Frailty (SCARF) index^23^. Data on co-morbidity and frailty were considered missing for patients without a hospital admission during the 2-year look-back period (for example, those whose first recorded admission occurred after the date of CRC diagnosis). HES data were also used to determine ethnicity and socioeconomic deprivation. Deprivation was captured using quintiles of the national distribution of the Index of Multiple Deprivation in Lower Layer Super Output areas (or neighbourhoods), with a typical population of approximately 1500 people and 600 households^24^.

#### Characteristics of NHS hospitals

Within the English NHS, hospital-level care is provided by hospital trusts. A trust is an organizational unit that can include more than one hospital. In this paper, the term ‘hospital’ refers to a hospital trust. The 2022 NBOCA organizational survey includes detailed information on the facilities available at every NHS hospital that provides care for patients with CRC^25^. This includes information about services such as perioperative review for elderly patients and the on-site availability of a high-risk MDT. NHS hospitals were further characterized based on whether they were a comprehensive cancer centre (that is, offering cancer surgery, radiotherapy, and systemic therapies on-site within their hospital)^26^, the mean annual volume of patients diagnosed with stage II or III CRC at the hospital, and their geographical region (north, midlands, and south; *Table S1*).

#### Patient selection

Patients aged ≥ 18 years with a primary diagnosis of stage II or III CRC between 1 April 2017 and 31 March 2022 were identified in the NBOCA database. Patients who could not be linked to an HES record, were not diagnosed in the NHS (for example, private-sector hospitals), or had missing Index of Multiple Deprivation data were excluded.

#### Exposure and outcomes of interest

The primary exposure was age, and the primary outcome of interest was receipt of surgical resection. The association between age and receipt of surgery was examined across categories of PS, co-morbidities, tumour site, and mode of presentation. A patient was considered to have undergone surgical resection if either the NBOCA recorded a surgical resection or the HES recorded an OPCS-4 procedure code for resectional CRC surgery^27^ in the 6 months before to 12 months after the date of diagnosis. Where the NBOCA and HES date of surgery did not correspond, the surgery date closest to the date of diagnosis was used. The secondary analysis examined between-hospital variation in rates of surgical resection.

#### Statistical analysis

Patient, tumour, and hospital characteristics were compared using χ^2^ tests according to whether patients had undergone surgical resection. A multilevel multivariable logistic regression model, with patients nested within hospitals, was used to assess the receipt of surgical resection, accounting for patient, tumour, and hospital characteristics. Age was modelled as a restricted cubic spline with five knots and validated against a model categorizing age into 5-year bands^28^ (*Fig. S1*). It was considered clinically plausible that the association between surgical resection and age may differ across PS categories, RCS Charlson scores, tumour sites, and modes of presentation. Therefore, interactions between age and each of these characteristics were tested separately. The statistical significance of the interactions was formally tested with the likelihood ratio test. The impact of including each of these interactions was assessed visually by comparing the marginal predicted probabilities of surgical resection (and the 95% confidence interval (c.i.)) with and without the interaction term^29^.

Patients with missing data for PS, ethnicity, RCS Charlson score, or the SCARF index were included using the missing-indicator method^30^. Data was complete for all other variables. Because surgical resection was relatively common, the results of the regression analysis from the full models (including interaction terms found to be important) were used to calculate risk ratios using marginal standardization^31,32^. This method estimates marginal predictions (that is, the average predicted probability of receipt of surgical resection across the study population, applying the fitted model to the observed distribution of patient, tumour and hospital characteristics for each possible value of age).

When assessing between-hospital variation, only patient and tumour characteristics were included in the risk-adjustment model. Intraclass correlation coefficients (ICCs) were used to quantify the between-hospital variation in the rate of surgery, after risk adjustment for patient and tumour characteristics. ICCs were estimated separately for two age groups (patients aged ≤ 75 and > 75 years). The ICCs between the age strata were compared using an independent-samples *Z*-test with two-tailed *P* values.

Funnel plots were used to visualize between-hospital variation in the unadjusted and adjusted rates of surgical resection^33,34^. The unadjusted and adjusted surgical resection rates for each hospital were plotted against the total number of patients diagnosed with stage II or III CRC at that hospital. To derive the adjusted rate of surgical resection by hospital, the predicted probability of surgical resection for each patient was calculated from the fixed-effects part of the risk adjustment model and then summed to obtain the total expected number of resectional surgeries by hospital. For each hospital, the observed number of resectional surgeries was divided by this expected total and multiplied by the national crude rate. Two hospitals (The Christie NHS Foundation Trust and The Royal Marsden NHS Foundation Trust) had an observed annual mean volume of patients newly diagnosed with stage II or III CRC of less than ten and were not presented in the funnel plots. These tertiary cancer centres typically do not diagnose bowel cancer.

STATA^®^ version 17 (StataCorp, College Station, TX, USA) was used for all analyses.

## Results

**Fig. 1:**
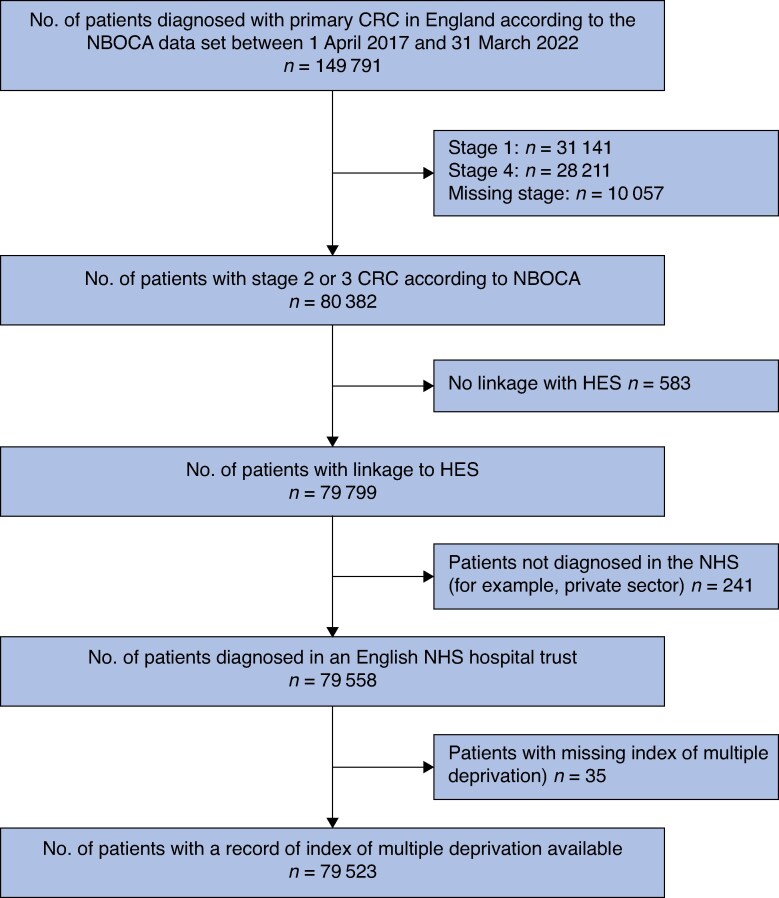
Flow chart showing inclusion of patients in the study CRC, colorectal cancer; NBOCA, National Bowel Cancer Audit; HES, Hospital Episode Statistics; NHS, National Health Service.

As shown in *Fig. 1*, 149 791 patients were diagnosed with CRC during the study period. Of these, 69 409 patients with stage I, stage IV, or missing stage, 583 patients identified in the NBOCA database without linkage to HES, 241 patients not diagnosed in the NHS (for example, private-sector hospitals), and 35 patients with missing Index of Multiple Deprivation data were excluded. Thus, 79 523 patients were included in the analysis. Patients with stage I disease were excluded because local excision is not fully captured in the data set.

Of the 79 523 patients newly diagnosed with stage II or III CRC in 133 hospitals included in this study, 63 489 (79.8%) underwent surgical resection. Baseline characteristics of patients and surgical resection rates are summarized in *Table 1*. The rate of surgical resection was fairly constant (at just below 90%) up to age 75 years, but decreased steeply in older patients, to 46.1% in those aged ≥ 85 years (*P* < 0.001). Surgical resection rates were also lower in patients from the most socioeconomically deprived quintile of neighbourhoods, and in those with poorer PS, more co-morbidities, a more severe level of frailty, tumours located in the rectum, and an emergency presentation.

**Table 1:** Distribution of patient, tumour, and NHS hospital characteristics overall and according to receipt of surgical resection

	All patients	Patients undergoing surgical resection	P*
Total no. of patients	79 523 (100%)	63 489 (79.8%)	
**Age group (years)**			< 0.001
< 50	4922 (6.2%)	4413 (89.7%)	
50–59	9541 (12.0%)	8432 (88.4%)	
60–74	32 278 (40.6%)	28 278 (87.6%)	
75–84	23 592 (29.7%)	18 130 (76.9%)	
≥85	9190 (11.6%)	4236 (46.1%)	
**Sex**			0.297
Male	44 486 (55.9%)	35 575 (80.0%)	
Female	35 037 (44.1%)	27 914 (79.7%)	
**Ethnicity**			< 0.001
White	72 016 (94.2%)	57 363 (79.7%)	
South Asian	1734 (2.3%)	1409 (81.3%)	
Black	1277 (1.7%)	1072 (84.0%)	
Other/mixed	1411 (1.8%)	1181 (83.7%)	
Not stated/not known	3085 (3.9%)	2464 (79.9%)	
**Index of Multiple Deprivation**			< 0.001
1st quintile (least deprived)	17 711 (22.3%)	14 371 (81.1%)	
2nd quintile	17 907 (22.5%)	14 484 (80.9%)	
3rd quintile	16 621 (20.9%)	13 321 (80.2%)	
4th quintile	14 312 (18.0%)	11 365 (79.4%)	
5th quintile (most deprived)	12 972 (16.3%)	9948 (76.7%)	
**ECOG PS**			< 0.001
0	36 682 (52.0%)	32 805 (89.4%)	
1	21 123 (29.9%)	17 325 (82.0%)	
≥ 2	12 727 (18.0%)	6491 (51.0%)	
Missing	8991 (11.3%)	6868 (76.4%)	
**RCS Charlson co-morbidity score‡**			< 0.001
0	41 546 (55.7%)	35 761 (86.1%)	
1	20 083 (26.9%)	15 883 (79.1%)	
≥ 2	12 999 (17.3%)	8270 (63.6%)	
Missing§	4895 (6.2%)	3575 (73.0%)	
**Frailty (SCARF index)**			< 0.001
Fit	28 226 (37.8%)	24 471 (86.7%)	
Mild frailty	20 803 (27.9%)	17 465 (84.0%)	
Moderate frailty	15 761 (21.1%)	12 213 (77.5%)	
Severe frailty	9838 (13.2%)	5765 (58.6%)	
Missing§	4895 (6.2%)	3575 (73.0%)	
**Tumour site**			< 0.001
Right-sided colon	31 763 (39.9%)	26 837 (84.5%)	
Left-sided colon	25 698 (32.3%)	21 696 (84.4%)	
Rectum	22 062 (27.7%)	14 956 (67.8%)	
**Clinical stage**			< 0.001
Stage II	29 917 (37.6%)	24 316 (81.3%)	
Stage III	49 606 (62.4%)	39 173 (79.0%)	
**Mode of presentation**			< 0.001
PCP or other HCP/screening	65 685 (82.6%)	53 240 (81.1%)	
Emergency admission	13 838 (17.4%)	10 249 (74.1%)	
**Year of diagnosis**			< 0.001
2017–2018	15 136 (19.0%)	12 363 (81.7%)	
2018–2019	15 656 (19.7%)	12 702 (81.1%)	
2019–2020	16 785 (21.1%)	13 417 (79.9%)	
2020–2021	14 801 (18.6%)	11 662 (78.8%)	
2021–2022	17 145 (21.6%)	13 345 (77.8%)	
**Hospital characteristics**			
Provision of perioperative Care of the Elderly service			0.002
No	15 408 (19.4%)	12 163 (78.9%)	
Yes	64 115 (80.6%)	51 326 (80.1%)	
High-risk MDT			0.014
No	10 361 (13.0%)	8178 (78.9%)	
Yes	69 162 (87.0%)	55 311 (80.0%)	
Comprehensive cancer centre			0.360
No	43 548 (54.8%)	34 716 (79.7%)	
Yes	35 975 (45.2%)	28 773 (80.0%)	
Annual hospital volume of patients diagnosed with stage II/III CRC			< 0.001
Low (< 100/year; 56 NHS hospitals)	19 937 (25.1%)	16 136 (80.9%)	
Medium (100 to < 150/year; 47 NHS hospitals)	28 303 (35.6%)	22 529 (79.6%)	
High (≥ 150/year; 32 NHS hospitals)	31 283 (39.3%)	24 824 (79.4%)	
Hospital region			< 0.001
North	25 462 (32.0%)	19 710 (77.4%)	
Midlands	20 765 (26.1%)	16 864 (81.2%)	
South	33 296 (41.9%)	26 915 (80.8%)	

Values are *n* (%). †In this study, ECOG PS was categorised as 0 (fully active), 1 (only able to carry out light or sedentary work), and ≥ 2 (no longer able to work or worse). ‡Number of co-morbidities. §Patients without a Hospital Episode Statistics hospital admission in the 2-year look-back period were defined as ‘missing’ for the RCS Charlson co-morbidity score and SCARF index. NHS, National Health Service; ECOG, Eastern Cooperative Oncology Group; PS, performance status. RCS, Royal College of Surgeons; SCARF, Secondary Care Administrative Records Frailty; PCP, primary care physician; HCP, healthcare professional; MDT, multidisciplinary team; CRC, colorectal cancer. *χ^2^ test.

Surgical resection rates were also slightly lower in patients diagnosed in the last 2 years of the study period (between 1 April 2020 and 31 March 2022, coinciding with the COVID-19 pandemic) and in patients who lived in the north of England.

None of the hospital characteristics (that is, perioperative review of elderly patients, on-site availability of a high-risk MDT, comprehensive cancer care, and annual hospital volume) was strongly associated with the rate of surgical resection.

### Multilevel modelling

**Fig. 2:**
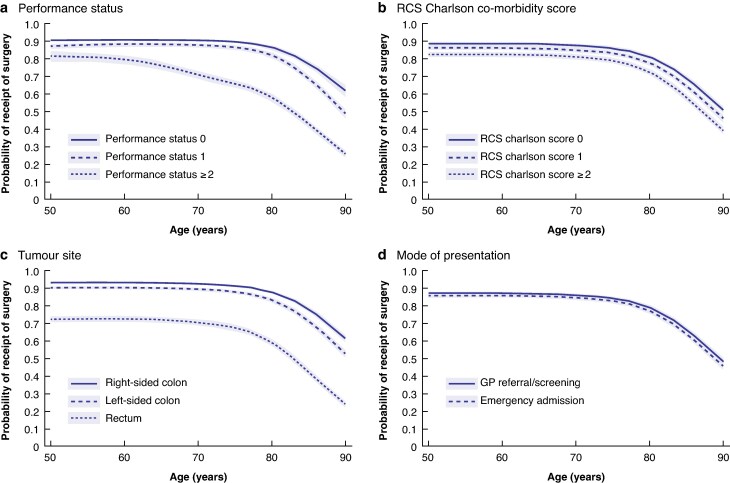
Adjusted predicted probabilities of receiving surgical resection according to age and performance status, co-morbidities, tumour site, and mode of presentation **a** Eastern Cooperative Oncology Group performance status (including an interaction between age and performance status), **b** RCS Charlson co-morbidity score, **c** tumour site, and **d** mode of presentation. Shaded areas indicate 95% confidence intervals. RCS, Royal College of Surgeons; GP, general practitioner.

*Figure 2* shows the relationship between age and the receipt of surgical resection by PS, RCS Charlson score, tumour site, and mode of presentation, adjusted for other patient, tumour and hospital characteristics.

All interactions were statistically significant (*P* < 0.001), but only the interaction with PS was included, because it substantially changed the association between surgical resection and age (*Fig. S2*). For patients with a PS of 0 or 1, the risk-adjusted rates of surgical resection remained constant, at around 90%, until age 75 years and then steeply declined by age 90 years to 61.8% (95% c.i. 58.3% to 65.3%) and 48.8% (95% c.i. 45.9% to 51.7%), respectively. However, for patients with a PS of ≥ 2, the risk-adjusted rate of surgical resection remained stable at around 80% until age 60 years and then gradually declined to 25.7% (95% c.i. 23.5% to 27.8%) at age 90 years (*Table S2*).

The adjusted relative risks for the rate of surgical resection using the full model (that is, the model including an interaction of age with PS) are presented in *Table 2*. Model interaction terms are presented in *Table 3*.

**Table 2:** Relative risks of receipt of surgical resection by patient, tumour, and National Health Service hospital characteristics

	Adjusted relative risk	*P*
**Sex**		
Male	1 (Reference)	0.073
Female	0.99 (0.99, 1.00)	
**Ethnicity**		
White	1 (Reference)	< 0.001
Asian	0.98 (0.96, 1.01)	
Black	0.98 (0.96, 1.01)	
Other/mixed	0.99 (0.96, 1.01)	
Not stated/not known	0.95 (0.94, 0.97)	
**Index of Multiple Deprivation**		
1st quintile (least deprived)	1 (Reference)	< 0.001
2nd quintile	0.99 (0.98, 1.00)	
3rd quintile	0.98 (0.97, 0.99)	
4th quintile	0.97 (0.96, 0.98)	
5th quintile (most deprived)	0.95 (0.94, 0.96)	
**RCS Charlson co-morbidity score**		
0	1 (Reference)	< 0.001
1	0.97 (0.96, 0.97)	
≥ 2	0.91 (0.90, 0.92)	
Missing	0.88 (0.87, 0.90)	
**Frailty (SCARF index)**		
Fit	1 (Reference)	< 0.001
Mild frailty	1.00 (0.99, 1.01)	
Moderate frailty	0.99 (0.98, 1.00)	
Severe frailty	0.92 (0.91, 0.94)	
Missing	0.88 (0.87, 0.90)	
**Tumour site**		
Right-sided colon	1 (Reference)	< 0.001
Left-sided colon	0.96 (0.95, 0.96)	
Rectum	0.73 (0.71, 0.74)	
**Clinical stage**		
Stage II	1 (Reference)	< 0.001
Stage III	0.96 (0.95, 0.96)	
**Source of referral**		
GP referral/screening	1 (Reference)	< 0.001
Emergency admission	0.98 (0.97, 0.99)	
**Year of diagnosis**		
2017–2018	1 (Reference)	< 0.001
2018–2019	0.99 (0.99, 1.00)	
2019–2020	0.97 (0.96, 0.98)	
2020–2021	0.96 (0.95, 0.97)	
2021–2022	0.94 (0.93, 0.95)	
**Perioperative review by Care of the Elderly**		
No	1 (Reference)	0.398
Yes	1.01 (0.98, 1.05)	
**High-risk MDT**		
No	1 (Reference)	0.354
Yes	1.02 (0.98, 1.07)	
**Comprehensive cancer centre**		
No	1 (Reference)	0.979
Yes	1.00 (0.97, 1.03)	
**Hospital volume**		
Low (< 100/year)	1 (Reference)	0.205
Medium (100 to < 150/year)	0.98 (0.95, 1.01)	
High (≥ 150/year)	0.97 (0.94, 1.01)	
**Hospital region**		
North	1 (Reference)	0.027
Midlands	1.05 (1.01, 1.09)	
South	1.02 (0.99, 1.05)	

Values in parentheses are 95% confidence intervals. RCS, Royal College of Surgeons; SCARF, Secondary Care Administrative Records Frailty; GP, general practitioner; MDT, multidisciplinary team.

**Table 3:** Model interaction terms

	Adjusted relative risk				*P*
	PS 0	PS 1	PS ≥ 2	PS missing	
PS (age 70 years)	1 (Reference)	0.97 (0.96–0.98)	0.78 (0.76, 0.80)	0.92 (0.90, 0.94)	< 0.001
**Age (years)**					< 0.001
50	1.00 (0.99, 1.01)	0.99 (0.98, 1.01)	1.15 (1.10, 1.20)	1.04 (1.02, 1.07)	
60	1.00 (0.99, 1.01)	1.01 (0.99, 1.02)	1.12 (1.09, 1.16)	1.03 (1.01, 1.05)	
70	1 (Reference)	1 (Reference)	1 (Reference)	1 (Reference)	
75	0.99 (0.99, 1.00)	0.99 (0.98, 0.99)	0.93 (0.92, 0.94)	0.96 (0.95, 0.97)	
80	0.95 (0.94, 0.97)	0.93 (0.92, 0.95)	0.82 (0.79, 0.85)	0.86 (0.83, 0.88)	
85	0.85 (0.84, 0.87)	0.79 (0.77, 0.81)	0.60 (0.58, 0.63)	0.65 (0.63, 0.68)	
90	0.68 (0.65, 0.72)	0.56 (0.53, 0.59)	0.36 (0.34, 0.39)	0.40 (0.37, 0.45)	

Values in parentheses are 95% confidence intervals. In this study, Eastern Cooperative Oncology Group PS was categorised as 0 (fully active), 1 (only able to carry out light or sedentary work), and ≥ 2 (no longer able to work or worse). PS, performance status.

### Variation between hospitals

**Fig. 3:**
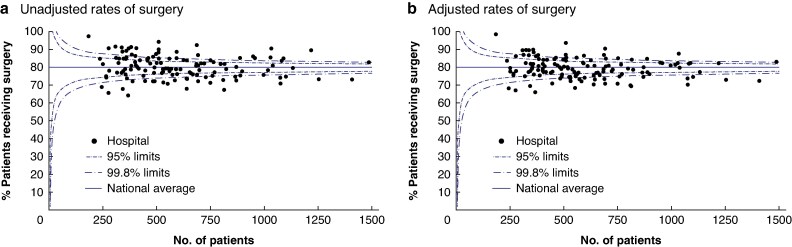
Funnel plots showing rates of surgery for patients with stage 2 or 3 colorectal cancer according to English National Health Service hospital with 95% and 99.8% funnel limits **a** Unadjusted and **b** adjusted rates of surgery for patients with stage II or III colorectal cancer. Risk adjustment was made for patient and tumour characteristics, including age, sex, ethnicity, Index of Multiple Deprivation, Eastern Cooperative Oncology Group performance status, the number of co-morbidities, frailty, tumour site, clinical stage, mode of presentation, and year of diagnosis. An interaction between age and performance status was included in the risk-adjustment model.

**Fig. 4:**
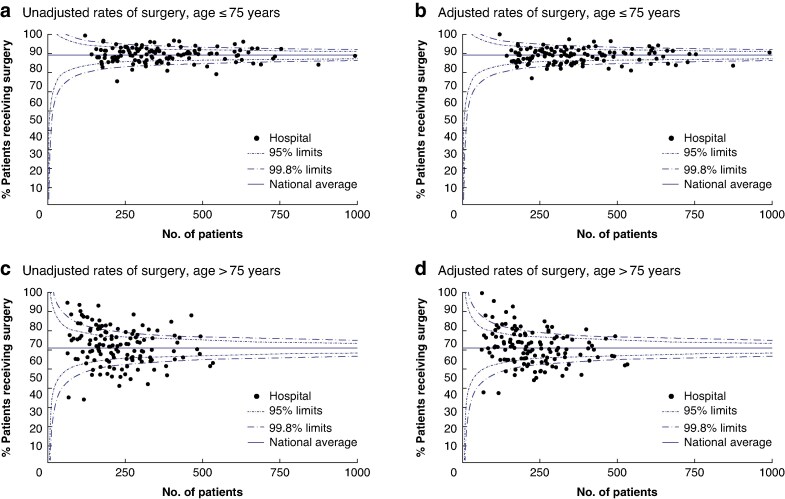
Funnel plots showing the rates of surgery for patients with stage 2 or 3 colorectal cancer according to English National Health Service hospital and age **a** Unadjusted and **b** adjusted rates of surgery in patients aged ≤ 75 years; **c** unadjusted and **d** adjusted rates of surgery in patients aged > 75 years. Risk adjustment was made for patient and tumour characteristics, including age, sex, ethnicity, Index of Multiple Deprivation, Eastern Cooperative Oncology Group performance status, the number of co-morbidities, frailty, tumour site, clinical stage, mode of presentation, and year of diagnosis. An interaction between age and performance status was included in the risk-adjustment model.

There was substantial variation among the 133 hospitals in surgical resection rates, and this variation changed little with risk adjustment. The interquartile range (i.q.r.) for the adjusted surgical resection rate by hospital was 75.5–83.0%. After risk adjustment, 48 hospitals (36.1%) had rates outside the 99.8% funnel plot limits, compared with 0.3 hospitals expected by chance alone (*Fig. 3*). Variation in the receipt of surgical resection was much greater among older patients. The i.q.r. of the adjusted surgical resection rate by hospital ranged from 60.3% to 73.7% in patients over 75 years of age and from 83.6% to 90.0% in those aged ≤ 75 years. After risk adjustment, 40 hospitals (30.1%) were outside the 99.8% funnel plot limits in those aged > 75 years. This compares to 23 hospitals (17.3%) outside the 99.8% funnel plot limits in those aged ≤ 75 years (*Fig. 4*). Of the 23 hospitals outside the 99.8% limits in patients aged ≤ 75 years, 14 (61%) were also outside the 99.8% limits in patients aged > 75 years.

The ICC, quantifying the between-hospital variation in the risk-adjusted rates of surgical resection for patients aged ≤ 75 years, was 0.051 (95% c.i. 0.038 to 0.068), compared with 0.111 (95% c.i. 0.086 to 0.141) for patients aged > 75 years (*P* < 0.001), confirming that the between-hospital variation was larger in older patients.

## Discussion

This study accurately quantified surgical resection rates in patients with newly diagnosed CRC by age and by measures of patient fitness. PS had the greatest impact on this association. Of the patients who were relatively fit (that is, those who are fully active or able to perform light or sedentary work), surgery was performed in around 90% up to age 75 years, with a steep drop in the surgery rate above this age. In patients who were less fit (that is, would no longer be able to work), the rate of surgery declined earlier, with approximately 80% undergoing surgery up to age 60 years, followed by a more gradual decline. These results demonstrate that treatment recommendations in the English NHS overall seem to be based on age and patients’ fitness, as recommended by the current clinical guidelines^3,17,35^. However, there is wide variation between hospitals in surgical decision-making, particularly in older patients.

The results of the present study are consistent with those of earlier studies^36,37^. The lower rates of surgery in older and less fit patients may reflect the greater perceived physiological insult of surgery overall in older and less fit patients, as well as the higher risks of postoperative mortality and specific complications, including anastomotic leaks^38,39^. The lower rate of surgical resection for rectal cancer may also reflect the greater impact of a permanent stoma in older patients^40^. The growing emphasis on enhanced surveillance and organ-preservation strategies following a complete clinical response to neoadjuvant therapies may further explain these age- and fitness-related findings^41^. Patients diagnosed after 2018 were also less likely to undergo surgical resection. This trend was not reversed after the COVID-19 pandemic, which may also reflect an increased use of organ-preservation strategies^42^. Patients with a complete clinical response following neoadjuvant therapies cannot currently be identified in the data set, but future research should track the evolution of rectal cancer management strategies, such as organ preservation in older patients.

Substantial between-hospital variation in the surgical resection rate persisted after risk adjustment for patient and tumour factors. This variation was more pronounced in patients > 75 years of age, which likely reflects greater clinical uncertainty in older patients^43^.

Addressing this age-related variation between hospitals requires a multifaceted approach. Research on practice variation highlights the importance of quality assurance in improving the quality of clinical care, especially for older patients^44^. Public reporting of surgical resection rates across English hospitals could help identify disparities and guide targeted interventions to improve uptake, not only for CRC patients overall but also for specific age groups. At the same time, efforts to reduce this variation in surgical rates in older patients must balance the immediate risk of surgical resection against its longer-term benefits.

The on-site availability of services within hospitals, including perioperative review of elderly patients, a high-risk MDT, and whether patients were treated in a comprehensive cancer centre, was not found to have a substantial impact on surgery rates. However, these findings should be interpreted with caution, given that data on these characteristics were reported by the hospitals, and details on the implementation, fidelity, and sustainability of these services are lacking^45^. It nevertheless raises the possibility that eligible patients may not always have access to these services, which, according to evidence in other cancer settings (for example, lung cancer), may benefit treatment decision-making in older patients^45–47^. An alternative explanation is that centres providing these services may manage a more complex or higher-risk patient cohort that is not fully accounted for within the risk-adjustment framework.

Despite guidelines recommending that treatment decisions be based on fitness rather than age, and advocating preoperative optimization of co-morbidities and risk stratification of high-risk patients using cardiopulmonary exercise testing^3^, substantial between-hospital variation persists in risk-adjusted rates of surgery, particularly among older patients. This suggests that current guidelines may not have translated into consistent standardization of care. Potential limitations of current guidelines include the absence of standardized multidisciplinary risk-stratification frameworks (involving surgeons, clinical nurse specialists, anaesthetists, and geriatricians), with defined thresholds to support shared decision-making. In addition, limited public reporting of guideline adherence and surgical resection rates may further contribute to unwarranted variation.

These findings demonstrate the importance of considering standardized treatment algorithms in elderly patients incorporating evidence-based strategies and shared decision-making. These may involve preoperative cardiopulmonary exercise testing or the 400-m 6-minute walk test to assess perioperative risk^48,49^, as well as a comprehensive geriatric assessment to evaluate frailty, as recommended by Getting It Right First Time^11,50^. Preoperative optimization and prehabilitation programmes have also been recommended in older and less fit patients to reduce the risk of major complications^51^. For example, NHS hospitals have implemented multidisciplinary preoperative assessments for older or less fit patients involving clinical nurse specialists, anaesthetists, and geriatricians to discuss risks and provide tailored recommendations for optimization and perioperative care^52^. All these initiatives would contribute to reducing unwarranted between-hospital variation in the receipt of surgical resection for newly diagnosed CRC patients while preventing unintended negative consequences for older patients.

Using representative national linked data sets, the relationship between age and rates of surgical resection was flexibly modelled in a large cohort in this study, describing the interplay between measures of physical fitness and age on surgical decision-making.

A key limitation is that the rates of surgical resection could only be studied, and not whether surgical resection had been offered. However, the variation in resection rates between hospitals was substantial, and evidence from breast cancer patients suggests that lower surgery rates in older patients are unlikely to be fully explained by patient choice^53^. The linked available national data sets do not have data items recording how decisions were made and reasons why surgical resection was not performed. For example, several factors that may have influenced treatment decisions, such as patient preference, cardiopulmonary exercise testing results, and social support, were not captured in the data set. These unmeasured variables may account for some of the variation between hospitals.

A further limitation of the study is that within the scope of this work, the effectiveness of surgical resection by age in terms of survival, complications, and quality of life was not estimated. For example, changes in social circumstances as a result of undergoing surgery or managing a stoma can be particularly challenging for those with co-morbidities such as impaired vision, cognitive decline, or osteoarthritis. Discharge destination would also have been useful as a surrogate marker of those patients who had potentially experienced unintended harms by undergoing surgery.

Missing data are a problem in most national data sets. Clinical stage was missing in 8.3*% of the 79 523 patients included in this study and was substituted with a recorded pathological stage. Because preoperative staging investigations are routinely performed before surgical resection, missing clinical stage in this group is most consistent with incomplete data capture. Across the entire cohort (149 791 patients), 77 149 patients had both clinical and pathological stage recorded: 85.3% of patients with pathological stage II or III cancer had clinical stage II or III cancer, indicating that patients with pathological stage II–III cancer were unlikely to have had clinical stage I or IV disease. Although clinical tumour staging demonstrates good accuracy for T3–4 categories, it remains limited for N categories^54^, introducing potential misclassification between stages II and III in the group of patients with substituted pathological staging. However, the receipt of surgical resection was similar in stage II (81.3%) and stage III (79.0%) cancer, limiting the potential impact of misclassification on the primary outcome.

Four patient characteristics had missing values, ranging from 3.9% missing ethnicity to 11.3% missing PS, and 19.6% of patients had missing values for at least one of these characteristics. All patients in the analysis were included, including those with missing data, by using the missing indicator approach^55^.

Overall, it was documented that in patients fit enough to work, age is not associated with the receipt of surgical resection for stage II–III CRC until after the age of approximately 75 years, when there is a steep decline in the receipt of surgery. However, in less fit patients, there is an earlier and more gradual decline in the receipt of surgery. Furthermore, substantial variation was found in surgical decision-making between hospitals, particularly in older patients. These findings demonstrate that providing evidence-based guidelines on assessing the appropriateness of surgical resection for high-risk CRC patients, linked to their age and physical fitness, has potential for improving the overall survival of patients with CRC and reducing variation between hospitals. There is also scope for surgical prehabilitation to reduce the risks of surgical resection in older and less fit patients.

## Supplementary Material

zrag071_Supplementary_Data

## Data Availability

The data that support the findings of this study are available from Healthcare Quality Improvement Partnership (HQIP). Restrictions apply to the availability of these data, which were used under licence for this study. Data are available from https://www.hqip.org.uk/national-programmes/accessing-ncapop-data/#.Y-ZRBK3P1hE with the permission of HQIP.
