# Supplementary material for: Association between age and surgical resection for patients with stage II or III colorectal cancer: national study
Source: BJS Open. 2026 Jun 11;10(3):zrag071. doi: 10.1093/bjsopen/zrag071 (PMC13255999; doi:10.1093/bjsopen/zrag071)
Supplement: zrag071_Supplementary_Data [file zrag071_supplementary_data.docx]

**Association between age and surgical resection for patients with stage II or III colorectal cancer: a national study**

Adil Rashid1,2, Helen Blake3, Lu Han1,2, Jan van der Meulen1,2, Michael S Braun4,*, Nicola S Fearnhead5*, Kate Walker1,2*

^1^Clinical Effectiveness Unit, Royal College of Surgeons of England, London, UK.

^2^Department of Health Services Research and Policy, London School of Hygiene and Tropical Medicine, London, UK.

^3^Department of Applied Health Research, University College London, London, UK.

^4^Department of Oncology, The Christie NHS Foundation Trust, School of Medical Sciences, University of Manchester, Manchester, UK.

^5^Department of Colorectal Surgery, Cambridge University Hospital NHS Foundation Trust, Cambridge, United Kingdom.

**Corresponding author.** Adil Rashidm adil.rashid1@lshtm.ac.uk **ORCID ID**; **Twitter** Mr_AdilRashid

**Supplementary Materials - Index**

| **Supplementary Figures and Tables** |  |
| --- | --- |
| Supplementary Table 1 Geographic region classified by Cancer Alliance. | *page 3* |
| Supplementary Figure 1 Risk-adjusted marginal predicted probabilities of receipt of surgical resection by age | *page 5* |
| Supplementary Figure 2 Adjusted predicted probabilities of receiving surgical resection by age, with and without an interaction between age and (a) performance status, (b) RCS Charlson score, (c) tumour site, (d) mode of presentation | *page 6* |
| Supplementary Table 2 Risk-adjusted predicted probability of receipt of surgical resection derived from the “full model” stratified by performance status, RCS Charlson Score, tumour site, and source of referral | *page 7* |

**Supplementary Figures and Tables**

Supplementary Table 1 Geographic region classified by Cancer Alliance

| Geographical region | Cancer Alliance |
| --- | --- |
| North | Cheshire and Merseyside  Greater Manchester  Humber, Coast and Vale  Lancashire and South Cumbria  Northern  South Yorkshire and Bassetlaw  West Yorkshire and Harrogate |
| Midlands | East Midlands  East of England (North Cancer Alliance)  West Midlands |
| South | East of England (South Cancer Alliance)  Kent and Medway  North Central London  North East London  Peninsula  RM Partners  Somerset, Wiltshire, Avon & Gloucestershire  South East London  Surrey and Sussex  Thames Valley  Wessex |

Cancer Alliances—regional structures that coordinate cancer-care pathways within 21 defined geographical areas.

*NHS England. Cancer alliances—improving care locally.* [*https://www.england.nhs.uk/cancer/cancer-alliances-improving-care-locally/#priorities*](https://www.england.nhs.uk/cancer/cancer-alliances-improving-care-locally/#priorities)

Supplementary Figure 1 Risk-adjusted marginal predicted probabilities of receipt of surgical resection by age


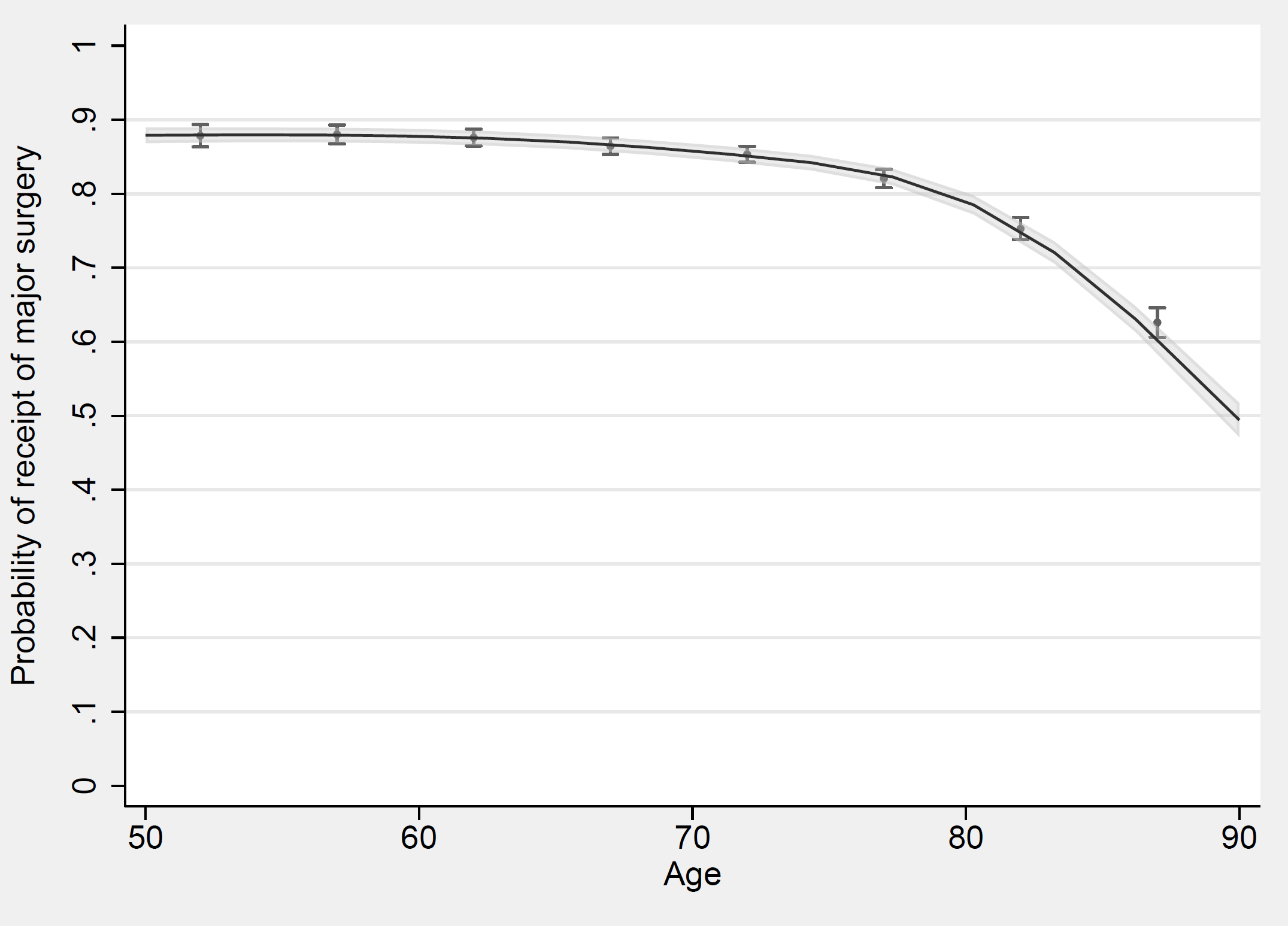


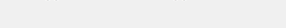


Probability of receipt of surgery

The black line represents the adjusted predicted probability of receipt of surgical resection derived from the multilevel multivariable logistic regression model, with age modelled as a restricted cubic spline. The grey shaded area indicates the 95% confidence interval. Superimposed symbols (with 95% confidence intervals) show the predicted probability from the same model with age categorised into 5-year bands, plotted at the median age within each band. Risk-adjustment includes all patient, tumour, and hospital characteristics.

*Supplementary Figure 2 Adjusted predicted probabilities of receiving surgical resection by age, with and without an interaction between age and (a) performance status, (b) RCS Charlson score, (c) tumour site, (d) mode of presentation*


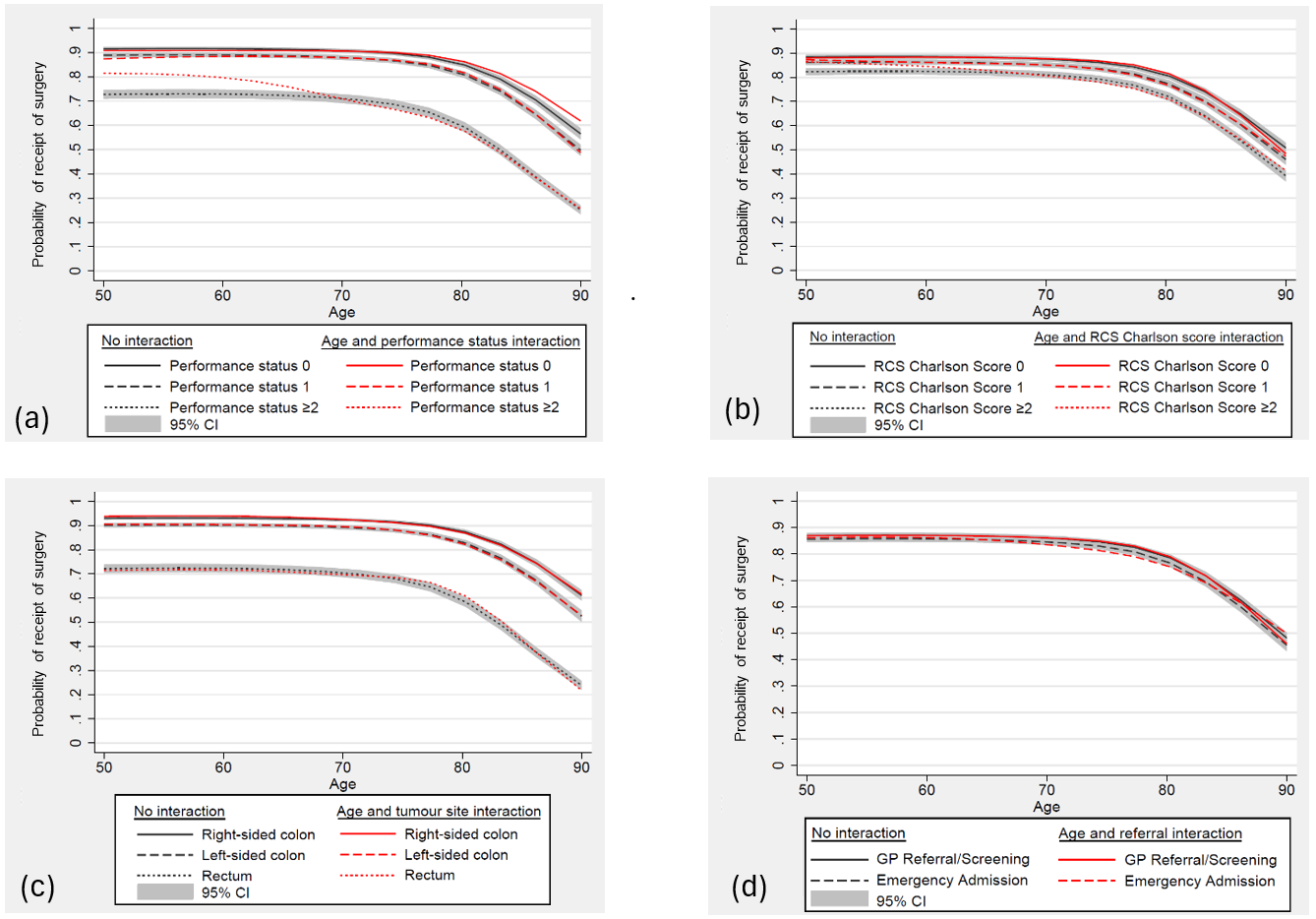


*Missing categories are not displayed*

Supplementary Table 2 Risk-adjusted predicted probability of receipt of surgical resection derived from the “full model” stratified by performance status, RCS Charlson Score, tumour site, and source of referral

|  |  | Adjusted predicted probability of receipt of surgical resection with 95% confidence intervals | | | | |
| --- | --- | --- | --- | --- | --- | --- |
|  |  | 50 years | 60 years | 70 years | 80 years | 90 years |
| Performance status | 0 | 0.91 (0.90, 0.92) | 0.91 (0.90, 0.92) | 0.91 (0.90, 0.92) | 0.87 (0.85, 0.88) | 0.62 (0.58, 0.65) |
|  | 1 | 0.87 (0.86, 0.89) | 0.88 (0.87, 0.90) | 0.88 (0.87, 0.89) | 0.82 (0.81, 0.84) | 0.49 (0.46, 0.52) |
|  | ≥2 | 0.82 (0.78, 0.85) | 0.80 (0.77, 0.82) | 0.71 (0.69, 0.73) | 0.58 (0.56, 0.60) | 0.26 (0.24, 0.28) |
|  |  |  |  |  |  |  |
| RCS Charlson Score | 0 | 0.90 (0.89, 0.90) | 0.89 (0.88, 0.90) | 0.88 (0.87, 0.89) | 0.81 (0.80, 0.83) | 0.52 (0.50, 0.55) |
|  | 1 | 0.87 (0.86, 0.89) | 0.87 (0.86, 0.88) | 0.85 (0.84, 0.86) | 0.78 (0.77, 0.80) | 0.48 (0.45, 0.50) |
|  | ≥2 | 0.84 (0.82, 0.85) | 0.84 (0.82, 0.85) | 0.81 (0.80, 0.83) | 0.73 (0.71, 0.75) | 0.41 (0.39, 0.44) |
|  |  |  |  |  |  |  |
| Tumour site | Right-sided colon | 0.94 (0.93, 0.95) | 0.94 (0.93, 0.95) | 0.92 (0.92, 0.93) | 0.88 (0.87, 0.89) | 0.63 (0.60, 0.65) |
|  | Left-sided colon | 0.91 (0.90, 0.92) | 0.91 (0.90, 0.92) | 0.89 (0.88, 0.90) | 0.83 (0.82, 0.85) | 0.54 (0.52, 0.57) |
|  | Rectum | 0.74 (0.72, 0.76) | 0.73 (0.72, 0.75) | 0.71 (0.69, 0.72) | 0.60 (0.58, 0.62) | 0.26 (0.24, 0.28) |
|  |  |  |  |  |  |  |
| Source of referral | GP Referral/Screening | 0.88 (0.87, 0.89) | 0.88 (0.87, 0.89) | 0.86 (0.85, 0.87) | 0.79 (0.78, 0.81) | 0.50 (0.48, 0.52) |
|  | Emergency Admission | 0.87 (0.86, 0.88) | 0.87 (0.86, 0.88) | 0.85 (0.83, 0.86) | 0.78 (0.76, 0.79) | 0.47 (0.45, 0.50) |

*Missing categories not displayed*
